# Anabolic Androgenic Steroids—Use and Correlates among Gym Users—An Assessment Study Using Questionnaires and Observations at Gyms in the Stockholm Region

**DOI:** 10.3390/ijerph8072656

**Published:** 2011-06-29

**Authors:** Håkan Leifman, Charlotta Rehnman, Erika Sjöblom, Stefan Holgersson

**Affiliations:** 1 STAD, Stockholm Centre for Psychiatric Research and Education, Karolinska Institutet and Stockholm County Council Health Care Provision, Box 6031, 113 60 Stockholm, Sweden; E-Mails: charlotta.rehnman@sll.se (C.R.); erika.sjoblom@sll.se (E.S.); 2 Department of Management and Engineering (IEI), Linköping University, 581 83 Linköping, Sweden; E-Mail: stefan_holgersson@hotmail.com

**Keywords:** anabolic androgenic steroids, supplements, gyms, observations, prevention, questionnaire, Stockholm County, weight training

## Abstract

The purpose of this study was to estimate the prevalence of anabolic androgenic steroid (AAS) use and offers to use among gym users in Stockholm County (Sweden), and to conduct a comparison of concordance in estimates of AAS and supplements at gyms between two data collection methods. A questionnaire was distributed to members at 36 training facilities and 1,752 gym users participated in the study. An observation study was conducted as covert participant observations at 64 gyms. According to the questionnaire, 3.9% of men reported life time use of AAS, 1.4% use during the past 12 months and 0.4% AAS use during past 30 days. Not only were there similar patterns found in the two methods, *i.e.*, similar age and gender distributions for AAS use, but analyses of concordance showed that gyms with a higher prevalence of self-reported AAS-use and supplement use (questionnaire) showed a significantly higher proportion of observer-assessed AAS users. Analyses of individual predictors showed that AAS users were almost always young men, regular weight trainers and more often users of drugs and nutritional supplements. The higher prevalence of AAS use among gym users than in the general population makes the former an appropriate target group for AAS prevention. The connection between supplements, drugs and AAS use suggests that effective AAS prevention need to focus on several risk factors for AAS use. The clear resemblance in estimates between the observation and questionnaire data strengthen the credibility of the two methods.

## 1. Introduction

During recent years, the use of anabolic androgenic steroids (AAS) has received increased attention, not only as a doping issue inside, but also outside the elite world of sport [1–5]. In Sweden for instance, most specialists in the field would probably agree that most AAS users are non-elite sportsmen who for various reasons want to achieve a more muscular physique. Consequently, use of AAS is increasingly perceived of as a bigger societal problem.

Whereas alcohol and narcotic prevention research continues to develop and to improve, our knowledge of what distinguishes effective preventive methods or programs from less effective ones, AAS prevention is more or less a blank page at the moment. Since studies have shown a relationship between AAS use and use of other drugs (for an overview of previous studies, see [2]) one could perhaps argue that some alcohol and drug prevention also impacts on AAS by targeting risk factors common to all drug users. At the same time, however, AAS users are not identical to other drug users and/or alcohol misusers [1,5] suggesting that there may be a need to also develop and test prevention programs specifically targeting AAS use.

Since many AAS users are also regular gym members, this makes gyms a potentially important arena for reaching current, and perhaps more importantly, potential future AAS users for preventive purposes [6–8]. This combined with the health and social risks associated with steroid use [9] as well as some good experiences in Stockholm of community-based alcohol and drug prevention programs on licensed premises [10,11] we developed and implemented a community AAS intervention program in the Stockholm region. The main goal of the intervention program was to reduce the use of AAS and reduce the availability of these drugs among visitors to gym and fitness centres in the Stockholm county. This included several prevention components, the most important being co-operation with important local actors and stakeholders (*i.e.*, the gym centres, local authorities [police, municipality]), training of gym staff and policy work at the gyms. The aim of the interventions was to reduce the use of AAS (and growth hormone) among the gym users (members and casual visitors) at gyms and to reduce the availability of steroids in these venues. Running from late 2007 with ongoing work planned until 2011, the research project included several phases, starting with a prevalence and observation base-line study in autumn 2007 and early 2008, to assess the extent of steroid use in Stockholm gyms. The program was implemented in 22 intervention gyms in Stockholm, and the effects of the intervention over time were (and still are) compared with control gyms in where no interventions are conducted. A follow-up prevalence survey to assess the impact of the intervention on AAS use was recently conducted (spring 2010) and a follow-up observation study were be conducted in the autumn of 2010.

The main aim of this study is, firstly, to estimate the prevalence of AAS use and AAS offers to test and buy, and secondly, to study correlates or predictions of AAS use/offers among gym users, based on both the base-line questionnaire and observation study conducted 2007 and 2008. Thirdly, since two data collection methods are used, a comparison of concordance in estimates between the two will also be conducted.

In the first part of the Results section, the questionnaire results will be presented, followed by findings from the observation study and then, lastly, the comparison of the two data collection methods. As far as we know, this is the first time two data sources are used in order to assess the AAS use and correlates/predictors of AAS use among gym users. This kind of triangulation of data is even more important when there is little previous knowledge of AAS at gyms and, further, when there is no established data collection method.

## 2. Material and Methods

### 2.1. Inclusion Criteria

Initially, an invitation letter to participate in the intervention study was sent out to all training facilities (gyms) in Stockholm County listed in the yellow pages, in total 180. All those that were interested to participate were selected to be part in the study as intervention gyms, altogether 22 gyms. Thereafter 22 gyms were matched to the 22 intervention gyms as control gyms. The matching was done according to geographical location, category of gym sport facilities (see next paragraph), the gyms’ price level and number of members.

Included in the intervention project (thus both intervention and control gyms) were public gyms which offer weight training facilities in the Stockholm region (*i.e.*, Stockholm County). The gyms in both data collections represent different categories of gym sport facilities such as sport centres, private gyms and gym chains. Several different areas in Stockholm County are represented in the study, both city and suburb. The gyms included in this study also vary in the number of members and size.

The observation study was also conducted in additional gyms not included in the intervention project; they were thus neither interventions nor control gyms. Some of the gyms in this group were strength-oriented gyms, so-called “hard core gyms”. The reason for the inclusion of these latter gyms was to get at more comprehensive picture of AAS use at the gyms in Stockholm County. The same inclusion criteria were used for all gyms.

### 2.2. Questionnaire Study

During the autumn 2007, 36 gyms in our intervention study participated in the baseline study (22 intervention gyms, 14 control gyms [in eight control gyms it was not possible to conduct the questionnaire study]) with the aim to assess the prevalence of steroid use and related problems (availability, drug use, use of supplements etc.) among people training at these gyms. The participating gyms were thus not randomly selected but were those that choose to be included in the, still ongoing, AAS prevention intervention study. Based on the number of gyms in Stockholm County at that time, the 36 gyms amounted to approximately 20 percent of the gyms that could be eligible to the program.

The questionnaire consisted of 41 questions and included background variables, training intensity (including weight training), use of supplements, narcotics and alcohol habits. Further, some attitude items focusing on AAS preventive measures were included. The first page on the questionnaire provided general information about the study.

The questionnaire was distributed at each of the gyms on two separate occasions, the first occasion being a weekday (between 16–21 hours), and the other day being a Saturday or Sunday (between 13–16 hours). All members visiting the gyms during these two days and hours were given the questionnaire either after or before they had been training in the weight lifting room. It should be mentioned that the questionnaire was distributed only to those training with weights (free weights or machines) at the actual gym, not to those taking part in aerobic training or other types of exercise at the gym.

In order to guarantee anonymity, the gym users were asked to fill in the questionnaire and then to place it in an envelope and to seal it. For non Swedish-speaking members, an English version of the questionnaire was available. A minimum of completed questionnaires at each gym and visit was set at 20. Although the task was to ask all gym users visiting the venues at the days and hours when the questionnaires were handed out, it is possible that a few gym users were missed.

In total, 123 subjects declined to participate, the most common reasons being ‘lack of time’, ‘not interested’ and ‘did not want to’. Another 108 subjects were given the questionnaire to be completed at home and then to send it back later in sealed envelopes. The reason for this was lack of time to fill in the questionnaire at that particular day and time. Fifty eight of them did complete the questionnaire and returned it to the project address. These 58 respondents showed a significantly higher proportion of women and a higher mean age than the other respondents but no significant difference in AAS use and AAS offers, controlling for gender and age.

In total, 1,752 gym users filled in the questionnaire and, thus another 173 (123 + 50) did not. Consequently, the non-response rate reached 9.0 percent (173/[1,752 + 173]). The internal non-response rate varied, but seldom reached 2–3 percent.

### 2.3. Observation Study

The data was collected as part of a covert participant observation study at 64 gyms, including all but two of the gyms in the questionnaire study. It also included an additional 30 gyms not in the questionnaire study. The observers were given a list consisting of 64 gyms but with no information whether the gyms belonged to the intervention group, the control group or to the group of the remaining 30 gyms. The observation instrument used in this study included 20 items. Some variables in the observation instrument focused on the number of machines, free weights, if the gyms were selling supplements, if the gyms had a policy against steroid use and if an instructor was present in the gym at the time for the observation. Others were estimated by the observer.

The following variables were observed and recorded for each gym user (and gym instructor): gender, approximate age (age classes: >20, 20–24…, 55–59, 60+) and estimation of likelihood of AAS use (according to the following seven degree scale: 1 = It is obvious that the individual uses AAS; 2 = It is very likely that the individual uses AAS; 3 = It is likely that the individual uses AAS; 4 = It would not be surprising if the individual used AAS; 5 = It is neither likely nor unlikely that the individual uses AAS; 6 = It is not likely that the individual uses AAS; 7 = It is very unlikely that the individual uses AAS). Thus the observers were required to make estimation of how likely it was that the gym user used AAS based on what he/she could observe at that particular time, mainly by looking at body characteristics which indicate the likely use of AAS: body constitution, body size, extent of acne, scars tissues but also appearance, particularly aggressive behaviour. To enhance the quality of the observation instrument, several pilot tests were conducted, four at sport facilities in Stockholm County and five at sport facilities in other regions in Sweden, representing different categories of sports facilities. None of these gyms were included in the actual study. After the pilot tests some minor changes were made to the instrument.

A research leader, experienced in observation studies, was recruited for the data collection. The research leader recruited three assistants and trained them in the observation instrument mentioned above. The training consisted of several pilot observations in different categories of sports facilities. In addition, a reliability test of the assessment of AAS use was conducted by letting the research leader and the assistants individually look at 12 pictures of different gym users of which some were users of AAS and others non-users and then score them individually from 1–7 according to the above scale. The internal consistency was rather high (Cronbach alpha = 0.89). Directly after this test of internal consistency, the results, and particularly the differences in assessment were discussed, in order to improve the consistency even further.

Naturally, the proportion of AAS users increases with wider assessment criteria used in the analyses of the seven degree scale. Besides analysing the assessment scale as a continuous variable, three different categorisations were used below. The strictest categorisation used (Criteria A) classified all gym users assessed as “obvious” and “very likely” AAS users (*i.e.*, the values 1–2 in the seven degree scale) and the remaining (values 3–7) as non-users. In the second categorisation, all gym users with values 1–3 were classified as AAS-users and the remaining as non-users, and in the least strict definition all gym users assessed within in the range 1–4 were classified as AAS-users, and the remaining as non-users.

On each of the 64 gyms, the research leader and the assistants were participating actively in training in order to observe the gym users who trained in the gym using the observation instrument for data collection. The observations were conducted at two occasions for each gym; one on a weekday (between 15–21 hours), the other at a Saturday or Sunday (between 9–17 hours) making a total of 128 observation occasions. Each time the observers spent approximately one hour at the gym and the actual observation period was 30 minutes. The observers dressed in the dressing room and trained in the weight lifting room. At the first occasion there were always two observers present, one of them always being the research leader. Since most gym environment observations were collected during the first gym visit, and they were not changed between the two time periods, the research leader was sometimes not supported by an assistant during the second visit. Below, data from both observation periods are included in the analyses of the assessments of the gym users. If a gym user was present at both observations occasions, however, he/she was only counted once, namely the first assessment. Still, it is possible that a few gym users were counted twice.

The research leader and the assistants used a training diary in which the observational instrument was included to fill in all information during the observations. Some information, however, was filled in directly after the visit was completed.

### 2.4. Comparing Questionnaire and Observation Data

The assessment of the degree of agreement between the two data sources is based on an aggregate level comparison, that is on the gym level, and focuses on estimates/assessment of AAS use and supplement use/sales on each gym (from the two data collections). Also the proportion offered AAS at each gym according to the questionnaire was compared to the proportion at each gym assessed as likely AAS users according to the observation study. All these analyses are based on the gyms that were included in both the questionnaire and the observation study, in total 34 gyms.

### 2.5. Statistical Tests

All tests of statistically significant differences in Tables 1–7 are based on chi-square tests, but when the expected values < 5, the Fisher’s Exact Test are used (for 2 × 2 tables or collapsed into 2 × 2 tables). The degree of concordance between results from the questionnaire and the observation study in Tables 8–9 are calculated as correlation coefficients based on Spearman’s rank correlation. Logistic multivariate regression analyses were conducted in order to test the impact of different explanatory variables controlling for other. Due to few numbers of AAS-users past 12 months and past 30 days, these analyses (not presented in any table) were only done only on life time use of AAS as well as on AAS-offers as outcome variables.

## 3. Results

### 3.1. Questionnaire

#### 3.1.1. Demographics

The mean age was 33 years (median = 29 years) among the 1,183 men and 38 (median = 35 years) among the 563 women. Fifty six percent among the men and 44 per cent among women were living as single (including living with parents), the remaining (men: 44%, women: 56%) were married or cohabiting. Compared to the general population in Stockholm County, a higher proportion of gym members are men, the average age is lower, and a higher proportion are not married/cohabiting. Not surprisingly then, a higher proportion than in the population are students. These and other relevant socio- demographics are also presented below in Table 2.

#### 3.1.2. Use of AAS

The proportions men and women reporting lifetime use of AAS, use during the past 12 months, and during the past 30 days are shown in Table 1. Just below 4 percent of the male gym members (45 men) reported having ever used AAS compared to 0.2 percent women (1 woman). AAS use during the past 12 months and past 30 days was much lower: 1.4 and 0.4 per cent among men. No woman reported AAS use during these two time periods. The proportion of AAS users per gym gives rather similar rates: men = 3.7% (life-time), 1.2% (past 12 months) and 0.4% (past 30 days).

Since only one woman reported use of AAS, the remaining analyses of AAS use concerns men only. Table 2 shows the proportion of male AAS users in different sub-groups. It should be kept in mind that among the men surveyed, relatively few reported any AAS at all, and in different subgroups the numbers become even smaller. Some patterns, however, are worth mentioning.

Respondents that were living alone reported higher 12 month and 30 day prevalence rates compared to those married/cohabiting. Students-secondary school or universities-reported lower levels of AAS use (life time and past 12 months) than the others (*i.e.*, those at work, unemployed, on sick leave, being pensioners and others). Furthermore, frequent binge drinkers reported higher life time prevalence than less frequent binge drinkers. The same pattern was found of users of narcotics and supplements: narcotic users reported significantly higher prevalence of life-time AAS use compared to non-users and the proportion of AAS users increased with increased frequency of supplement use.

Finally, those with the very highest frequency of weight training—10 hours or more per week or 5 times a week or more—showed higher prevalence of life time AAS use compared to the others. However, it should be mentioned that there were no statistically significant differences between the other frequency categories. For example, those training 7–9 hours/week reported no higher use of AAS compared to those training 4–6 hours/week.

Logistic regression analyses (not presented in any table) with life time use of AAS as the outcome (dependent) variable and the other variables in Table 2 as explanatory (independent) variables (percent concordant = 79.5; percent disconcordant = 18.7; Somer’s D = 0.607) showed significant effects of frequent weight training (≥3–4 times a week), (odds ratio = 6.6), narcotic use (used past 12 months) (odds ratio = 5.3) and use of supplements (≥twice a week) (odds ratio = 2.89). The remaining variables in table 2 turned out to be non-significant. No logistic regression analyses were conducted with AAS during past 12 months and past 30 days as dependent variable due to too few cases.

#### 3.1.3. Offered AAS

Several questions focused on the perceived availability of AAS, by asking the respondents whether or not they had been offered to buy AAS and if they had been offered to test AAS. As for using AAS, the proportions were much higher for men than for women, but for both sexes higher than for the prevalence of AAS use. This is evident from Table 3 which shows gender specific prevalence rates in different subgroups for the combined outcome measure of offers to buy AAS and/or to test AAS.

There were still too few women reporting being offered AAS (3.7%) in order to conduct more detailed analyses. Thus, logistic regression analyses (not shown in any table) were conducted only for men. Taken together, the results resembled the results of AAS use as the outcome variable mentioned above. Thus, narcotic use (past 12 months), frequent weight training (≥3–4 times a week), use of supplements (≥twice a week) show the strongest relationship with AAS offers. These three variables, together with frequent binge drinking (≥every week), age (age group 25–29 year-olds compared to the others) and employment status (those at work and others [unemployed, sick leave, pensioners] with higher levels than two student categories) remained as statistically significant with AAS offers (buy and/or test) as outcome variable and all eight variables in Table 3 as independent variables.

#### 3.1.4. Use and Offers of AAS in Different Categories of Gym

Three broad categories of gyms were represented in the questionnaire study. Table 4 shows the AAS distribution across these three types of gyms for men and women separately. The highest prevalence rate was found in private gyms and the lowest in gyms in sport centres but no differences, except one, were statistically significant. The exception was offers of AAS among men which were significantly more common in private gyms.

### 3.2. Observation Study

In total 2,368 individual gym users were assessed on their age, gender and particularly AAS use at the 64 gyms included in this study. Sixty nine per cent were men and 33 per cent women with the same ratio in the 34 gyms present in both studies (n = 1,281, thus 34 of the 36 intervention and control gyms). Two gyms in the questionnaire study were thus not included in the observation study. The 34 gyms are hitherto also named the questionnaire gyms.

Each gym user observed was age-classified according to five year intervals: ≤19, 20–24, 25–29…54–59, and ≥60 years of age. Eighteen per cent were assessed as 19 years of age or below, 40 per cent as 20–29 years of age, and 11 per cent as 35–39 years of age. Thus 75 per cent were classified as 35–39 or younger. The 34 questionnaire gyms, showed a higher proportion of the youngest (≤19 years of age) and a lower proportion of 25–29 year olds compared to the remaining 30 non-questionnaire gyms. In both groups the median age group was 26–30 years. The gender and age distribution according to the observation data accords quite well with the questionnaire data reported above.

#### 3.2.1. Assessment of proportion AAS users

Naturally, the proportion of assessed AAS users increases with wider assessment criteria, as shown in Table 5. In total, 1.6 per cent men (average per gym: 1.3%) were assessed as AAS users using the strictest definition (criteria A–obvious and very likely users–according to the seven grade scale), 4.9 per cent (gym average: 4.6%) with a less strict (category B–criteria 1–3) and 13.7 (gym average: 13.1%) with the least strict definition (category C–criteria 1–4). The rates were somewhat lower for the 34 questionnaire gyms (averages for all respondents: 1.0%, 3.9% and 11.8% respectively; gym averages: 0.7%, 3.2% and 10.2% respectively). In other words, those 30 additional gyms included in the observation study showed significantly higher proportions: 2.3%, 6.1% and 15.9% respectively (gym average: 2.0%, 6.1% and 16.4% respectively).

Category B (*i.e.*, the proportion on each gym that were given the assessment score 1–3: obvious, very likely, likely users) show the most similar prevalence compared to the life-time prevalence in the questionnaire study whereas category A (score 1–2) show the most similar rates as regards used 12 months according to the questionnaire.

In Table 6 the distribution of observer-assessed AAS use is shown by age group. According to category B and C, the 25–29 years of age shows the highest proportion followed by the age group 30–34. In category A, the 30–34 years old shows the highest proportion, followed by the age group 35–39 and 25–29. Thus, for all three assessment categories, the highest proportion is found in the age groups 25–29, 30–34 and 35–39. This holds true regardless of whether all gyms or only the questionnaire gyms are analysed.

Differences in the proportion of AAS assessed male users in different categories of gyms are shown in Table 7. One category of gyms stands out with significantly higher rates than the other three categories, namely so called “hard core” gyms. According to category B, almost 21% of all observed gym users at “hard core” gyms were assessed as likely AAS users compared to 6% in private gyms, roughly 4% in gym chains and almost 2% in gyms at sports centres. Among the 34 questionnaire gyms no “hard core” gyms were included and only four private. The differences among these three categories, however, differed significantly with highest rates found in private gyms and lowest among gym chains.

The observation data also entails information on whether or not the gyms were selling supplements. As shown in Table 8, the gyms that sell supplements show on average higher proportion (mean) of observation-assessed probable AAS users. The relationship becomes somewhat stronger if only the 34 questionnaire gyms are included in the analyses.

#### 3.2.2. Concordance between Results from the Questionnaire and the Observation Study

As was indicated above, a rather similar pattern was found in both the questionnaire and the observations: similar age and gender distribution, rather similar estimated AAS prevalence and a relationship between supplements and AAS use.

Here we look more closely at this relationship by studying the factual correlation between the gym questionnaire data and observational data on aggregate level gym data, thus only including the gyms that participated in both data collections (n = 34). If there is a correlation between questionnaire estimates of AAS use and AAS offers and observation-assessed AAS use, it strengthens the validity of the two methods.

As shown in table 9, most correlations are in the expected direction indicating a correlation between the two data collection methods in assessments/estimates. As regards AAS use, the gyms with a higher proportion of self-reported users (questionnaire) also show a higher proportion (mean) of observed-assessed likely users (Spearman’s rank correlation r_s_ = 0.51). Also, the gyms with a higher proportion of self-reported offers of AAS (according to the questionnaire) shows a significant relationship with observed-assessed AAS use (r_s_ = 0.47). Since use or availability of supplements was assessed in both studies (self-reported use and whether or not the gyms were selling supplements) they were included in our analyses. As shown in Table 9, gyms selling supplements were significantly more likely to also have respondents in the questionnaire reporting use of supplements (r_s_ = 0.57).

## 4. Discussion

According to the questionnaire data, roughly 4 percent of men reported a life time use of AAS (ever used), 1.4 per cent recent use (during the past 12 months) and 0.4 current use (during past 30 days). Only one woman reported ever used AAS, none current or recent use. The observations that were conducted at the questionnaire gyms (n = 34) indicated similar rates. According to the strictest criteria A (score 1–2 out of seven) approximately 1.0 percent were assessed as AAS users (compared to 1.4 recent users in the questionnaire), and according to the middle criteria (B: score 1–3 out of seven) 3.9 percent of men were assessed as AAS users (compared to 3.9 percent life-time users in the questionnaire). According to the broadest criteria for use (C: score 1–4) 11.8 percent were assessed as likely AAS users. All rates from the observations were somewhat higher if all 64 gyms were included.

The observations were not conducted on the same days that the questionnaires were handed out. In other words, not all respondents in the questionnaire were included in the observations and *vice versa*. Most likely, some are included in both, but how large that proportion is remains unknown. Despite that, the two first criteria (A, B) showed similar rates as the life time and recent AAS use obtained in the questionnaire. This could be interpreted as a random, coincidental occurrence, but the relationship found on the aggregate level for the 34 gyms included in both the questionnaire and the observation study were positive and did not occur by chance. In other words, those gyms with a higher prevalence of self-reported AAS use (according to the questionnaire) showed on average a higher proportion of likely AAS users according to the observations. This indicates that both methods, to a certain degree, capture the same phenomena.

In addition to AAS use, it was also found that respondents training at gyms selling supplements to a significantly higher degree of reported use of supplements in the questionnaire than those respondents at gyms with no sales. This relationship cannot be used as a validation of the observations since it was obvious for the observers whether or not the gyms were selling supplements, but it strengthens the validity of the questionnaire estimates. A perfect fit was not to be expected since it was not obvious that the respondents bought their supplements at the gyms they were visiting at the time of the data collection.

According to the Swedish Doping Act (SFS 1991:1969), with the exception of medical or scientific purposes, *i.e*., sales, use and possession of these drugs are considered as criminal acts. If the crime is considered to be misdemeanour, the person will be sentenced to fines or imprisonment of a maximum of six months. Upon a serious violation, the person will be sentenced to jail for a minimum of six months to a maximum of six years. This could have contributed to an increased non-response rate and to a social desirability in the responding to these questions of using AAS, which may have led caused to an underestimation of the actual rates. On the other hand, it cannot explain the very similar prevalence rates obtained in the two methods.

To sum up, not only are there similar patterns found in the two methods, i.e. similar age and gender AAS distributions, similar prevalence rate of AAS and similar links between supplements and AAS, but there were also significant correlations on the gym level in assessments of AAS and of supplements. In other words, gyms with a higher prevalence of self-reported AAS-use and supplement use (questionnaire) showed a significantly higher proportion of observer- assessed AA users and were more likely to sell supplements. This supports the contention that the observation data can be further analysed in order to enhance our understanding of the relationship between AAS use among gym members and different characteristics in the gym environment. The similar results also suggest that observation studies could an alternative to questionnaire studies in estimating prevalence of AAS use at gyms. However, more comparative studies of the two methods are needed in order to verify this.

Prevalence rate of approximately 5–6 percent among male gym users have been found in three other gym questionnaire studies in Sweden that we are aware of. One was conducted at gyms in the city of Kalmar [12] (5% men reported life time use), another at gyms in Malmö city [13] with 6% of men reporting ever used AAS, and the third in the county of Kronoberg with approximately 5.6 % of life time users among men [14]. Since the data collection procedure differs somewhat, including selection of gyms and respondents, they are not fully comparable with our estimates (of roughly 4% male life-time users). Despite this, however, the rates must be interpreted as rather similar in all four studies with 4–6% life-time AAS users among men and, generally speaking, very few women.

The corresponding AAS prevalence rates in the general Swedish population are much lower. According to a recent review of the Swedish situation, the prevalence of AAS use in the general population during recent years was estimated at one per cent life time users among men, at the most, and very few women (almost zero per cent). Corresponding 12-months prevalence rates were considerably lower [15]. In another web-based general population study with questions of AAS use, frequency of weight training and use of supplements conducted by our team in 2007 [16], 7.0 per cent of men and 0.8 women in Stockholm County in the ages 18–50 reported that they had ever been offered AAS, growth hormone or any other similar doping preparation (AAS: men 5.3% and women: 0.0%. These are also substantially lower rates than those found among gym users in the present study.

It should also be mentioned that despite a perceived increase in prevalence of AAS use in society, the reported levels above are considerably lower compared to other drugs such as narcotics, minor tranquillisers and alcohol. For instance, general population studies in Sweden suggest a life time prevalence rate of narcotics at 14–15 per cent for men and 7–8 per cent for women [17].

Internationally, the prevalence rate of AAS use at gyms has been shown to vary to a great extent, perhaps due to cultural differences in AAS and differences in legislation on doping between countries but probably also due to different types of gyms included in different studies which makes the comparability of the results obtained from different countries somewhat difficult. In any case, where comparisons have been made, the prevalence estimates at gyms have been shown to be considerable higher than the corresponding estimates in the general population [6,18,19]. Our study is thus no exception to this rule.

Turning to individual predictors of AAS use in the questionnaire data, (without necessarily implying causality), our results showed that AAS users (and those being offered AAS) were almost always young men, and most of them frequent weight trainers. They were also more often drug users. Several other studies have shown similar results, not the least a link between drug use and AAS use [2,6,20,21], important facts that should be taken into consideration when prevention interventions are developed. Thus, it is possible that drug prevention in general also has an effect on AAS use but also that AAS prevention should include drug prevention components.

Another of the factors strongest associated with AAS use in our analyses was supplement use, also this link has been shown in previous studies [6,8,20,21]. Perhaps one could say that, although supplements (and drugs) are certainly neither a sufficient nor a necessary cause for AAS it may increase the risk of using AAS. That is, using one type of preparation in order to enhance ones performance and/or appearance could make the step to another illegal preparation somewhat easier. The temptation of using supplements to improve athletic performance or even to enhance appearance by getting more muscles could be very seductive to young people, who are eager for quick results. Therefore, it is important to show young people in particular, the alternatives to the use of dietary supplements and providing them with realistic objectives and expectations with their training. This is also worth noticing when forming future AAS prevention methods.

Finally this study showed, not surprisingly, that AAS use is more common among gym users than among the general population, and, perhaps more important is that a substantial number of gym users have been offered AAS. Thus, gym users are at heightened risk of AAS use and therefore represent an appropriate target group for AAS prevention with gyms as a natural arena for these activities.

## Figures and Tables

**Table 1:** Number and proportion respondents reporting use of AAS during life-time, past 12 months and past 30 days, among men and women.

	Ever used AAS (life time)		Used AAS during past 12 months		Used AAS during past 30 days	
	n	%	n	%	n	%
Men (n = 1161)	45	3.9	16	1.4	5	0.4
Women (n = 560)	1	0.2	0	--	0	--

Differences between men and women: life time: *P < 0.001* (Chi-square: 19.85; df = 1); past 12 months: *P = 0.005* (Chi-square: 7.80; df = 1); Past 30 days: *P = 0.180* (Fisher’s Exact Test)), internal non-response gender: n = 6; use of AAS: n = 25, together: n = 31

**Table 2:** Number and proportion (%) men reporting use of AAS, life-time, past 12 months and past 30 days, in different subgroups.

	Ever used AAS (life-time)		Used AAS during past 12 months		Used AAS during past 30 days	
	n	%	n	%	n	%
*Age:*		NS		NS		NS
16–24 (n = 430)	12	2.8	7	1.6	3	0.7
25–29 (n = 157)	10	6.4	5	3.2	2	1.3
30–39 (n = 212)	11	5.2	2	0.9	0	0.0
40–49 (n = 166)	7	4.2	1	0.6	0	0.0
50+ (n = 183)	4	2.2	0	0.0	0	0.0
*“Marital status”*:		NS		S		NS
Single (n = 643)	24	3.7	13	2.0	5	0.8
Married/cohabiting (n = 497)	21	4.2	2	0.6	0	0.0
*Education*:		NS		NS		NS
Completed compulsory school (n = 255)	13	5.1	5	2.0	2	0.8
Completed secondary school (high school) (n = 586)	23	3.9	9	1.5	3	0.5
Completed university/college (n = 298)	7	2.4	2	0.7	0	0.0
*Employment*:		S		NS		NS
Student—secondary school (n = 187)	2	1.1	0	0.0	0	0.0
University/college student (n = 92)	1	1.1	1	1.1	0	0.0
At work (n = 772)	33	4.3	13	1.7	5	0.7
Other a (n = 95)	8	8.4	2	2.1	0	0.0
*Used Supplements:*		S		NS		NS
No use (n = 518)	9	1.7	5	1.0	2	0.4
Monthly or less often (n = 114)	3	2.6	3	2.6	1	0.9
Daily or weekly (n = 472)	30	6.4	7	1.5	1	0.2
*Binge drinking of alcohol:*		S		NS		NS
Never (n = 352)	11	3.2	3	0.9	0	0.0
Monthly or less often (n = 670)	20	3.0	7	1.1	3	0.5
Daily or weekly (n = 115)	9	7.9	3	2.6	1	0.9
*Used narcotics during past 12 months*		S		NS		NS
No (n = 1,036)	28	2.7	10	1.0	3	0.3
Yes (n = 929	12	13.0	2	2.2	0	0.0
(No answer [n = 32])	5	15.2	4	12.5	2	6.2
*Weight training:*		S		S		NS
Never or less than 1–2 times/week (n = 344)	3	0.9	1	0.3	1	0.3
3–4 times a week (n = 660)	24	3.7	9	1.4	3	0.5
5 times a week or more often (n = 175)	18	10.7	6	3.6	1	0.6
Total (n = 1161) b	45	3.9	16	1.4	5	0.4
Average per gym (n = 36 gyms)	--	3.7	--	1.2	--	0.4

a Unemployed (n = 33), sick leave (n = 14), pensioner (n = 26), others (n = 28);

b Altogether 45, 16 and 5 men reported life-time, past 12 months and past 30 days use of AAS, but due non-response the number for each variable do not always add up to 45; S = statistically significant, NS = not statistically significant; Age: 25–29 year olds versus other age groups—AAS past 12 months: *P = 0.055* (Fisher’s Exact Test); Marital status – AAS past 12 months: *P = 0.037* (Chi-square: 4.34; df = 1); Employment—AAS life time: ‘Other’ versus other employment groups: *P = 0.003* (Fisher’s Exact Test)); Binge drinkers—AAS life time: *P = 0.031* (Chi-square: 6.94; df = 1); Narcotic use—AAS life time: *P < 0.001* (Chi-square: 35.17; df = 1); Use of supplements—AAS life time: *P =< 0.001* (Chi-square: 14.87; df = 2); Weigh training—AAS life time: *P <0 .001* (Chi-square: 28.95; df = 2); AAS past 12 months: 5 times a week or more often versus other frequencies: *P = 0.020* (Fisher’s Exact Test).

**Table 3:** Proportion (%) reporting being offered AAS (offers to buy and/or offers to test) in different subgroups among men and women.

	Offered AAS	
	Men (n = 1,183)	Women (n = 563)
*Age:*	S	NS
16–24 (n = 441/n = 163)	23.8	6.8
25–29 (n = 163/n = 62)	38.0	4.8
30–39 (n = 213/n = 100)	31.0	3.0
40–49 (n = 168/n = 110)	18.4	1.8
50+ (n = 185/n = 123)	11.4	1.6
*“Marital status”*:	NS	NS
Single (n = 660/n = 247)	23.9	4.9
Married/cohabiting (n = 523/n = 316)	24.9	2.8
*Education*:	NS	S
Completed compulsory school (n = 262/n = 110)	21.0	8.2
Completed secondary school (high school) (n = 596 / n = 250)	26.3	2.4
Completed university/college (n = 300/n = 192)	23.3	3.1
*Employment*:	S	S
Student—secondary school (n = 196/n = 73)	16.3	13.7
University/college student (n = 92/n = 71)	25.0	0.0
At work (n = 778/n = 351)	26.6	2.3
Other^a^ (n = 101/n = 65)	23.8	4.6
*Used Supplements:*	S	NS
No use (n = 524/n = 365)	15.3	2.7
Monthly or less often (n = 115/n = 37)	25.2	5.4
Daily or weekly (n = 477/n = 126)	34.8	5.6
*Binge drinking of alcohol:*	S	NS
Never (n = 352/n = 282)	19.0	3.6
Monthly or less often (n = 670/n = 245)	24.3	4.1
Daily or weekly (n = 115/n = 18)	42.6	5.6
*Used narcotics during past 12 months*	S	NS
No (n = 1049/n = 520)	21.7	3.3
Yes (n = 94/n = 19)	56.4	10.5
*Weight training:*	S	NS
Never or less than 1–2 times/week (n = 344/n = 361)	14.0	2.5
3–4 times a week (n = 660/n = 169)	25.3	5.3
5 times a week or more often (n = 175/n = 32)	41.7	9.4
Total (n = 1183/563)	24.3	3.7
Average per gym (n = 36 gyms)	23.2	3.4

a Unemployed, sick leave, pensioner, others; S = statistically significant, NS = not statistically significant; Age—men: *P < 0.0001* (Chi-square: 41.87; df = 4)—women: 16–24 year olds versus other age groups: *P = 0.025* (Fisher’s Exact Test); Education—women: completed compulsory school versus other educational groups: *P = 0.011* (Fisher’s Exact Test); Employment—men: *P = 0.029* (Chi-square: 8.99; df = 3)—women: students-secondary school versus other working groups/students: *P < 0.001* (Fisher’s Exact Test); Binge drinkers—men: *P < 0.0001* (Chi-square: 26.05; df = 2); Use of supplements—men: *P < 0.001* (Chi-square: 51.33; df = 2); Narcotic use—men: *P < 0.001* (Chi-square: 55.86; df = 1); Weight training—men: *P < 0.001* (Chi-square: 49.05; df = 2).

**Table 4:** Number and proportion (%) men reporting use of AAS life-time, past 12 months and past 30 days, in different subgroups, in different gym categories.

	Gym chains (5 gyms)		Gyms in sport centres (18 gyms)		Private gyms (13 gyms)	
	n	%	N	%	n	%
*Ever used AAS*:						
Men (n = 175/576/404)	6	3.4	17	3.0	22	5.4
Women (n = 104/242/207)	0	0.0	1	0.4	0	0.0
*Used AAS past 12 months*:						
Men (n = 174/578/401)	1	0.6	8	1.4	7	1.8
Women (n = 104/242/207)	0	0.0	0	0.0	0	0.0
*Used AAS past 30 days*:						
Men (n = 174/578/401)	0	0.0	3	0.5	2	0.5
Women (n = 104/242/207)	0	0.0	0	0.0	0	0.0
*Offered AAS*:						
Men (n = 178/591/408)	41	23.0	121	20.5	126	30.9
Women (n = 104/244/208)	2	1.9	9	3.7	10	4.8

AAS-offers: men: *P = 0.001* (Chi-square: 14.38, df = 2). All other differences (AAS use and AAS offers for women) were not statistically significant.

**Table 5:** Number and proportion gym users (men and women) assessed as possible AAS users according to the observational instrument and different degrees of likelihood.

	Category A: score 1–2 (%) a		Category B: score 1–3 (%) a		Category C: score 1–4 (%) a	
*Sex*:	n	%	n	%	n	%
*All 64 gyms*^b^						
Men (n = 1642)	26	1.6	80	4.9	223	13.6
Women (n = 726)	2	0.3	3	0.4	10	1.4
*34 questionnaire gyms*^c^						
Men (n = 879)	9	1.0	34	3.9	103	11.8
Women (n = 402)	0	0.0	0	0.0	3	0.7

a 1 = It is obvious that the gym member use AAS; 2 = It is very likely that the gym member use AAS; 3 = It is likely that the gym member use AAS; 4 = It would not be surprising if the gym member use AAS; 5 = It is neither likely nor unlikely that the gym member use AAS; 6 = It is not likely that the gym member use AAS; 7 = It is very unlikely that the gym member use AAS);

b Differences between men and women: Category A: *P =< 0.007* (Chi-square: 7.4, df = 1), Category B: *P =< 0.001* (Chi-square: 25.6, df = 1), Category C: *P =< 0.001* (Chi-square: 84.5, df = 1);

c Category A: *P =< 0.0642* (Fisher’s Exact Test), Category B: *P =< 0.001* (Chi-square: 16.0, df = 1), Category C: *P =< 0.001* (Chi-square: 43.7, df = 1).

**Table 6:** Number and proportion gym users among men assessed as possible AAS users according to the observation study in different age groups.

	Category A: score 1–2 (%) a		Category B: score 1–3 (%) a		Category C: score 1–4 (%) a	
*Age groups:*	N	%	n	%	n	%
*All 64 gyms*^b^						
<20 (n = 305)	1	0.3	5	1.6	18	5.9
20–24 (n = 406)	2	0.5	14	3.4	77	19.0
25–29 (n = 264)	8	3.0	33	12.5	69	26.1
30–34 (n = 171)	9	5.3	17	9.9	40	23.4
35–39 (n = 110)	5	4.4	7	5.5	11	10.0
40–44 (n = 145)	1	0.7	4	2.8	7	5.0
45–49 (n = 105)	0	0.0	1	1.0	1	0.9
≥50 (n = 136)	0	0.0	0	0.0	0	0.0
*34 questionnaire gyms*^c^						
<20 (n = 218)	1	0.5	3	1.4	11	5.0
20–24 (n = 211)	1	0.5	7	3.3	49	23.2
25–29 (n = 97)	3	3.1	16	16.5	26	26.8
30–34 (n = 62)	4	6.5	7	11.3	13	21.0
35–39 (n = 54)	0	0.0	1	1.9	3	5.6
40–44 (n = 74)	0	0.0	0	0.0	1	1.4
45–49 (n = 67)	0	0.0	0	0.0	0	0.0
≥50 (n = 96)	0	0.0	0	0.0	0	0.0

a 1 = it is obvious that the gym user use AAS; 2 = It is very likely that the gym user use AAS; 3 = It is likely that the gym user use AAS; 4 = It would not be surprising if the gym user use AAS; 5 = It is neither likely nor unlikely that the gym user use AAS; 6 = It is not likely that the gym user use AAS; 7 = It is very unlikely that the gym user use AAS);

b, c All age differences for all categories were statistically significant (Chi-square tests or Fisher’s Exact Test, the latter based on a comparison between 25–34 year olds versus other age groups).

**Table 7:** Number and proportion gym users among men assessed as possible AAS users according to the observation study in different categories of gyms.

	Category A: score 1–2 (%) a		Category B: score 1–3 (%) a		Category C: score 1–4 (%) a	
	
	N	%	N	%	n	%
*All gyms (n = 64)*^b^						
Gym chains (n = 509)	8	1.6	22	4.3	67	13.2
Gyms at sport centres (n = 563)	2	0.4	10	1.8	42	7.5
”Hard core” gyms (n = 102)	8	7.4	21	20.6	43	42.2
Private gyms (n = 453)	8	1.8	27	6.0	71	15.7
*Questionnaire gyms (n = 34)*^c^						
Gym chains (n = 71)	0	0.0	0	0.0	3	4.2
Gyms at sport centres (n = 502)	2	0.4	9	1.8	38	7.6
”Hard core” gyms (n = 306)	--	--	--			
Private gyms (n = 453)	7	2.3	25	8.2	62	20.3

a see notes in Table 6;

b Category A: *P=< 0.001* (Chi-square: 30.9, df = 3), Category B: *P =< 0.001* (Chisquare: 66.9, df = 3), Category C: *P =< 0.001* (Chi-square: 90.0, df = 3);

c Category A: Private gyms versus other gyms: *P = 0.011* (Fisher’s Exact Test ), Category B: *P =< 0.001* (Chi-square: 23.9, df = 2), Category C: *P =< 0.001* (Chi-square: 33.8, df = 2).

**Table 8:** The relationship on aggregate gym level between selling supplements and the gyms’ mean score on assessed probability of AAS-use among all observed based on the observation data (n = 64/n = 34).

Selling suppleSments (Yes/No):	The gyms’ mean score on assessed probability of AAS use among gym users a	Spearman’s rank correlation (between supplement sales and assessed AAS use)
*All gyms (n = 64)*		
Supplements–yes	5.46	r^s^ = (−)0.37 (*P* = 0.030)
Supplements–no	6.00	
*Questionnaire gyms (n = 34)*		
Supplements–yes	5.76	r^s^ = (−)0.43 (*P* = 0.000)
Supplements–no	6.09	

a see notes in Table 6.

**Table 9:** The relationship on aggregate gym level between questionnaire and observation results regarding AAS use and offers and use and sales of supplements according to the 34 gyms included in both studies (n = 34).

	Questionnaire			Observation	
	AAS life time use	Offered AAS	Frequency of supplement use	Likely AAS users	Sales of supplements
*Questionnaire:*					
AAS life time use	--	0.48 (*P* = 0.003)	0.21 (*P* = 0.228)	0.51 (*P* = 0.002)	0.33 (*P* = 0.053)
Offered AAS	--	--	0.60 (*P* = 0.001)	0.47 (*P* = 0.005)	0.36 (*P* = 0.035)
Frequency of supplement use	--	--	--	0.50 (*P* = 0.003)	0.57 (*P* = 0.000)
*Observations*:					
Likely AAS users	--	--	--	--	0.43 (*P* = 0.000)
Sales of supplements	--	--	--	--	--
